# 1168. Cost-effectiveness of high-dose quadrivalent influenza vaccine versus standard-dose quadrivalent influenza vaccine for older people in a country with high influenza vaccination rate

**DOI:** 10.1093/ofid/ofad500.1008

**Published:** 2023-11-27

**Authors:** Eliel Nham, Hye Seong, Hakjun Hyun, Jin Gu Yoon, Ji Yun Noh, Hee Jin Cheong, Woo Joo Kim, Eugene Kim, Leejung Choi, Jung-Min Lee, Joon Young Song

**Affiliations:** Division of Infectious Diseases, Department of Internal Medicine, Korea University College of Medicine, Seoul, South Korea, Seoul, Seoul-t'ukpyolsi, Republic of Korea; Division of Infectious Diseases, Department of Internal Medicine, Korea University College of Medicine, Seoul, South Korea, Seoul, Seoul-t'ukpyolsi, Republic of Korea; Korea University Guro Hospital, Seoul, Seoul-t'ukpyolsi, Republic of Korea; Division of Infectious Diseases, Department of Internal Medicine, Korea University College of Medicine, Seoul, South Korea, Seoul, Seoul-t'ukpyolsi, Republic of Korea; Division of Infectious Diseases, Department of Internal Medicine, Korea University College of Medicine, Seoul, South Korea, Seoul, Seoul-t'ukpyolsi, Republic of Korea; Division of Infectious Diseases, Department of Internal Medicine, Korea University College of Medicine, Seoul, South Korea, Seoul, Seoul-t'ukpyolsi, Republic of Korea; Division of Infectious Diseases, Department of Internal Medicine, Korea University College of Medicine, Seoul, South Korea, Seoul, Seoul-t'ukpyolsi, Republic of Korea; Syneos Health Korea, Seoul, Seoul-t'ukpyolsi, Republic of Korea; Syneos Health Korea, Seoul, Seoul-t'ukpyolsi, Republic of Korea; Sanofi Korea, Seoul, Seoul-t'ukpyolsi, Republic of Korea; Division of Infectious Diseases, Department of Internal Medicine, Korea University College of Medicine, Seoul, South Korea, Seoul, Seoul-t'ukpyolsi, Republic of Korea

## Abstract

**Background:**

The high-dose quadrivalent formulation (QIV-HD) has shown improved protection against influenza and its complications in older adults. We aimed to evaluate the cost-effectiveness of QIV-HD compared with QIV-SD among Korean adults aged ≥ 65 years in reducing influenza-related disease burden.

**Methods:**

We evaluated the 2016/2017 and 2017/2018 seasons and their average values using a static decision tree model. For the main analysis, hospitalization was defined as cardiorespiratory disease-related. The difference in efficacy between standard-dose (SD) and high-dose (HD) was calculated based on the results of a clinical trial comparing Fluzone® High-Dose Vaccine and Fluzone® Vaccine in older adults. Incremental cost-effectiveness ratios (ICERs) were assessed from the healthcare system perspective. A discount rate of 4.5% was applied to life-year-gained (LYG) values and utilities. We performed deterministic and probabilistic sensitivity analyses to account for both epidemiological and economic sources of uncertainty.

**Figure:**
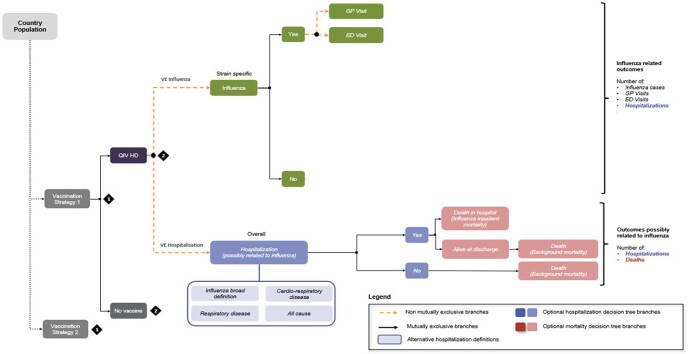

**Results:**

In the analysis of the 2017/2018 season, the QIV-HD strategy generated an excess of 0.003953 quality-adjusted life-years (QALYs) compared with QIV-SD. The ICER was 6,467.56 USD/QALY. For the 2016/2017 season, QIV-HD caused an excess of 0.003272 QALYs and ICER was 7,902.46 USD /QALY. From the average data of the two seasons, an excess of 0.003561 QALYs were generated and the ICER was 7,190.44 USD/QALY. In the one-way sensitivity analysis, the parameters with the greatest impact on the results were the relative VE of QIV-HD vs. QIV-SD against influenza-associated hospitalization. PSAs were conducted for the 2016/2017 and 2017/2018 seasons, with the cost of the QIV-HD vaccine fixed at 50,000 KRW (40.48 USD) or 65,000 KRW (52.62 USD). The results showed that for a WTP threshold of 24,692.36 USD/QALY, the probability that QIV-HD is a cost-effective option for the Korean health system is 100% in all seasons and QIV-HD vaccine costs considered.

**Figure:**
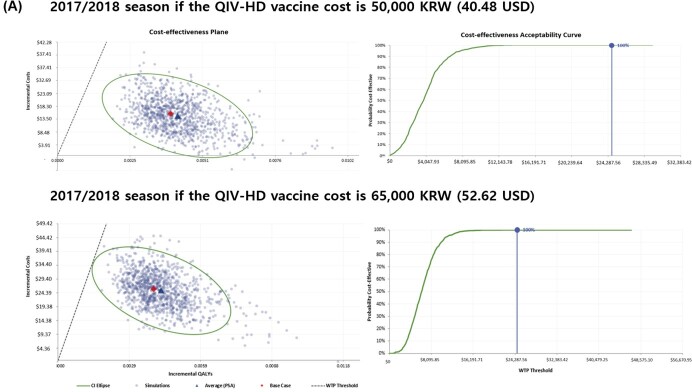

**Figure:**
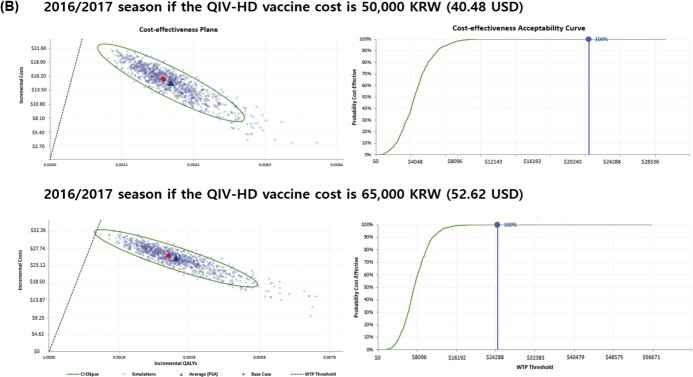

**Figure:**
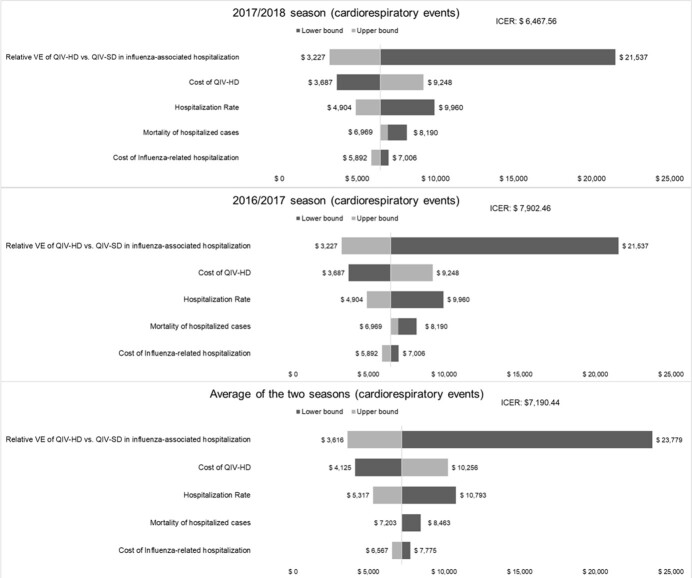

**Conclusion:**

From the healthcare system perspective, QIV-HD was a more cost-effective vaccination option in reducing influenza-related disease burden and healthcare costs in Koreans aged ≥ 65 years compared with QIV-SD.

**Disclosures:**

**Hee Jin Cheong, M.D., Ph.D.**, Sequiris: Advisor/Consultant **Jung-Min Lee, n/a**, Sanofi Korea: Stocks/Bonds

